# Assessing extreme sea level rise impacts on coastal agriculture in Europe and North Africa

**DOI:** 10.1038/s41598-025-31630-w

**Published:** 2025-12-20

**Authors:** Federico Martellozzo, Matteo Dalle Vaglie, Carolina Falaguasta, Filippo Randelli, Katarzyna Negacz, Pim van Tongeren, Bas Bruning, Pier Vellinga

**Affiliations:** 1 Department of Economics and Management (DISEI), University of Florence, Florence, Italy; 2 The Institute for Environmental Studies (IVM), Vrije Universiteit Amsterdam, Amsterdam, The Netherlands; 3 The Salt Doctors, Amsterdam, The Netherlands; 4 University of Rome La Sapienza, Dept. MEMOTEF, Rome, Italy

**Keywords:** Climate sciences, Environmental social sciences, Natural hazards

## Abstract

Sea Level Rise refers to the long-term increase of sea level. This phenomenon is primarily driven by the melting of ice caps and the thermal expansion of the oceans. Extreme Sea Level Rise (ESLR) events occur when SLR combines with temporary phenomena such as storm surges, tides, and waves, creating potentially damaging coastal flooding. The accelerating impact of climate change has raised the attention on ESLR and its effects on coastal regions. This study focuses on ESLR and its potential impacts on Europe and North Africa up to 2100, with particular attention to agriculture. Utilising Joint Research Centre (JRC) Global Extreme Sea Level projections and fine-scale DTM, we mapped areas vulnerable to ESLR under Representative Concentration Pathway (RCP) scenarios 4.5 and 8.5. Through a topological approach, we generated spatially explicit maps of at-risk regions. Then, the magnitude of ESLR’s impact on local agricultural systems was estimated by overlaying crop production data from FAO (GAEZ 2015+) with different flood scenarios. Findings reveal that ESLR can severely affect coastal agriculture, suggesting significant potential agricultural losses (from $800 million up to $1.5 billion per year in the next 100 years), impacting food security and economic stability. This research underscores the urgent need for adaptive strategies, including the construction of dykes and sea barriers and the shift of agriculture to salt tolerant crops to mitigate ESLR impacts.

**Supplementary Information:**

The online version contains supplementary material available at 10.1038/s41598-025-31630-w.

## Introduction

In recent years, the study of Sea Level Rise (SLR) has gained an unrivalled priority driven by the accelerating impact of climate change on our planet’s oceans^1^. The term SLR broadly refers to the gradual increase in the average level of planetary open water bodies^1,2^. Relative Sea Level Rise (RSLR) and Extreme Sea Level Rise (ESLR) are two critical concepts to this field, each representing a unique declination of the phenomenon^2^. While they are often discussed in tandem, their distinctions and interactions hold the key to understanding of the future of coastal environments and the broader implications for climate adaptation strategies^3^.

Relative Sea Level Rise (RSLR), often colloquially referred to as SLR, describes the change in sea level in relation to adjacent land surfaces^4,5^. This concept encapsulates both the absolute rise in global sea levels driven by the thermal expansion of seawater, the melting of ice sheets and local changes in land elevation^4^. Extreme Sea Level Rise (ESLR)^5^ as defined by the Intergovernmental Panel on Climate Change (IPCC), represents the upper limit of the projected sea level rise throughout the 21st century^6^. This upper limit is determined by amalgamating the highest estimates from all contributing factors with the additional effect of extreme events such as storms, surges, and high tides, which are predicted (with some degree of uncertainty) to become more severe and frequent in the future^5,6^. These extreme events, capable of causing catastrophic flooding, have become a focal point for researchers and policymakers, as they pose immediate and tangible threats to coastal communities and ecosystems^7^. The estimation for RSLR for the next ~100 years ranges from 0.2 to over 2 metres in the worst-case scenario^2,8^. The occurrence of an ESLR event can more than quadruple this value, potentially raising the sea level up to a maximum of 8 m^9^.

Global warming, primarily driven by the emission of greenhouse gases (GHGs), is the main cause of SLR. Increased GHG concentrations in the atmosphere lead to higher global temperatures, which in turn cause the melting of ice caps and the thermal expansion of ocean waters^2,7–9^. Recent research indicates that accelerated melting of the Greenland and Antarctic ice sheets may contribute more substantially to extreme sea level rise than thermal expansion alone, particularly under warming scenarios exceeding 1.5 °C^8,9^. Elevated temperatures also enhance the frequency and intensity of extreme weather events, including cyclones and storm surges, which amplify ESLR’s devastating effects on vulnerable coastal regions (e.g. higher risk of coastal flooding and erosion)^10^.

Overall, SLR, in both its Extreme and Relative forms, is poised to exert substantial impacts on global coastal regions, with the extent of these effects contingent upon various factors, including SLR rates, local infrastructure susceptibility, geomorphological characteristics, land use patterns, population growth trends, and community adaptive capacities^1,2,5,6,11^. While diverse sectors face vulnerability^11–14^, agriculture in low-lying coastal areas is particularly at risk^12^. Moreover, flooding in particular, can damage crops, reduce yields, and have long-term consequences for food security and economic stability^15–20^.

While we recognize that ESLR is not the only threat facing coastal agriculture, it is widely acknowledged in literature as one of the principal drivers of long-term land degradation, resulting in relevant productivity loss in low-lying regions. Hence, this work focuses on Extreme Sea Level Rise (ESLR) due to its potential to cause significant casualties and economic loss^21^. Our scope is to map areas vulnerable to ESLR in Europe and the Mediterranean basin to provide an initial assessment of the economic impacts on agriculture^22,23^. By considering different scenarios^3^ and vulnerability levels, we develop a comprehensive risk assessment to inform policy localization and effective mitigation strategies^22^. Unlike most studies that emphasize urban and infrastructure damage^24,25^, this research highlights the unique and enduring impacts of ESLR on croplands and food systems.

## Results

### Estimate ESLR vulnerability areas extent

The areas prone to ESLR in Europe and along the coast of North Africa are modelled making use of the Joint Research Centre (JRC) Global Extreme Sea Level projections, while the morphological analysis is based on DeltaDTM^26^ and AHN DEM (Actueel Hoogtebestand Nederland- Dutch National Elevation Dataset)^27,28^. The JRC defines ESLR as the sum of RSLR and Extreme Sea Levels (ESL) driven by tides, waves, and storm surges. All these factors are incorporated into both the Baseline Scenario, which is constructed using observed data from the period 1980–2014, and into four forward-looking climate projections that account for varying greenhouse gas emission pathways and timeframes. Specifically, the analysis explores two distinct climate change trajectories based on established Representative Concentration Pathways (RCPs): RCP 4.5, which represents a moderate emissions scenario assuming stabilization through mitigation efforts, and RCP 8.5, which reflects a high-emissions scenario consistent with limited or no climate policy intervention. These RCPs scenarios are used to model projected ESLR impacts under different and contrasting assumptions. The 4.5 RCP scenario conveys a moderate increase in global temperatures where the climate effect of human activities is dampened by effective mitigation actions^21^. Conversely, the RCP 8.5 is a more severe scenario in which emissions continue to rise throughout the twenty-first century. RCP 8.5 is generally used as the basis for worst-case climate change scenarios, it was initially based on what proved to be an overestimation of projected coal outputs^29^. The baseline scenario, derived from the work of Vousdoukas et al.^21^, represents present-day sea-level conditions for the reference period 1980–2014. During this period, tidal elevations along the global coastline were obtained using the FES2014 model, which combines tidal harmonics with ocean circulation and barotropic atmospheric forcing to generate a detailed depiction of current sea-level dynamics. This baseline provides the foundational reference for evaluating future impacts of Extreme Sea Level Rise (ESLR). By comparing future projections against this present-day benchmark, assessing changes in the extent and severity of coastal inundation under different climate scenarios is hence enabled. The two timeframes for which the effects of human activities are measured are 2050 and 2100, thus resulting in considering a total of 5 different scenarios For each scenario, three levels of inundation severity are considered, corresponding to the 5th, 50th, and 95th percentiles of the Extreme Sea Level Rise (ESLR) projections derived from Monte Carlo simulations, following the seminal work of Vousdoukas et al.^21^. These percentiles capture the range of potential outcomes, from a conservative lower-bound estimate at the 5th percentile, representing minimal sea level rise and limited inundation, through the 50th percentile, which reflects the median and most probable projection under current climate modelling assumptions, to the 95th percentile, which depicts a worst-case scenario with extreme sea level rise and widespread inundation. We assume the uncertainty as a proxy for vulnerability to generate spatial maps indicating the likelihood of specific regions being impacted by ESLR in the future^8,20^. In this work we assume “vulnerability” as the combination of geographical suitable conditions (i.e. low DEM—Digital Elevation Model—coupled with the span of probability of ESLR given the available projections).

The maps of the impacted areas are generated by subtracting the Digital Elevation Model (DEM) with the ESLR projections with 100-year return period. ESLR projections are presented as points on the coastline. Each point contains the sea level in metres for an extreme event that has the 1% possibility to occur each year. The projections intersect 5 climate scenarios and 3 *inundation severity classes* for a total of 15 possible combinations^30^. The spatial association of terrestrial locations with the nearest ESLR estimates along the coast, accomplished through Thiessen tessellation, identifies all raster inland elevation pixels associated with a specific ESLR sample value. Subsequently, subtracting elevation values from projected ESLR estimates allows for the determination of the geographical extent of regions likely to be impacted by future rising oceans^31^. The magnitude of the inundation was estimated by considering both the coastline distance and the slope of the terrain. Slope is indeed an accurate proxy for terrain hydrology^32,33^, allowing it to factor in floodwater pathways. Then, an exponential decay function is applied, and areas that don’t have pixels exhibiting spatial contiguity with the coastline or with permanent water bodies directly connected to it are identified as isolated and eliminated^32^. As a result, we obtain inundation maps in which each 30 m×30 m cell ranges from 0 to 100. This value points to the area of each cell being submerged and allows to create site-specific estimations of flood vulnerability.

### Historical insights and future projections

**Figure 1:**
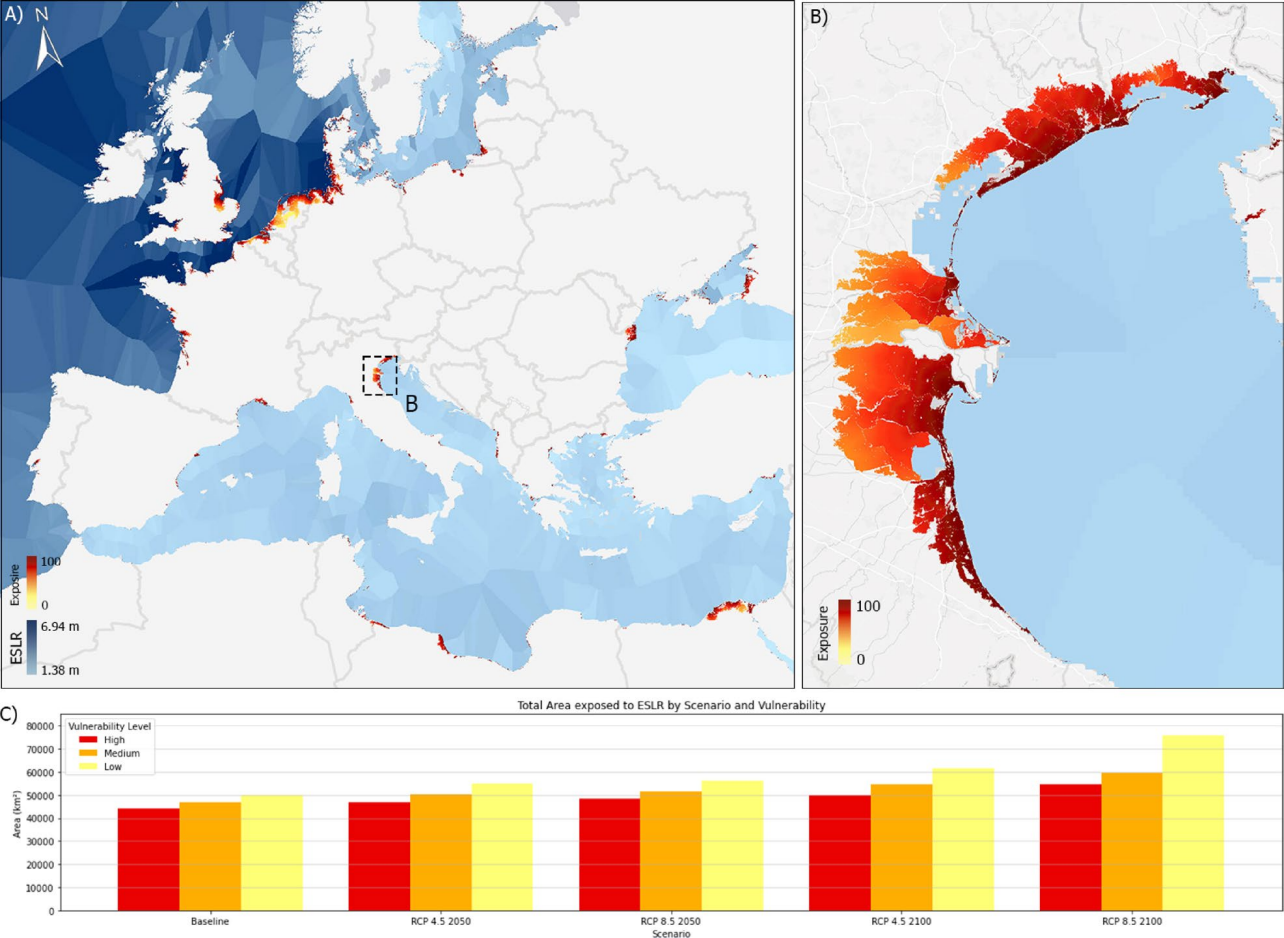
Area under the threat of an ESLR event and magnitude of the event with 100-year return period in 2100 RCP8.5 scenario (**A**). Zoom in the Po Delta region showing the medium vulnerability RCP8.5 2100 (**B**) scenario. Bar graph showing the total area vulnerable to ESLR in the different scenarios and for the three vulnerability levels (**C**). Map created by the authors using QGIS (3.40, url: https://qgis.org/)) and Python (3.15).

The first result stemming from our projections (Fig. 1) is that a higher exposure to ESLR is not linearly linked to a larger area of impacted land. This is due to the morphology and hydrology of the coastline. For example, in the west coast of Ireland ESLR projections are very severe but due to very high and steep cliffs the areas vulnerable to an extreme event are smaller^34^. On the contrary there are areas in which the magnitude of the projected extreme phenomenon is not among the most severe, but the consequences can be disastrous, such as in the Nile delta. Figure 1a outlines that there are 5 macro-areas showing a significant vulnerability to ESLR events. The first and most notable one groups the coasts of (i) Belgium, Netherlands, Germany, and Denmark facing the North Sea. This region has a long history of coastal flooding besides the already mentioned and famous North Sea Flood of 1953^35^ other events of similar magnitude have occurred over the centuries causing the spread of death and devastation. Notable among these are the St. Lucia’s flood of 1287, the St. Marcellus flood of 1362, the 1530 St. Felix’s flood, and the Christmas Flood of 1717^36^, each illustrating the lethal potential of North Sea storm surges. These events affected a broad swath of Northwest Europe, collectively resulting in approximately 14,000 deaths^35^. Many of these storm surges have also had disastrous consequences across the English Channel, identifying the (ii) United Kingdom as another high-risk area. Following this are the (iii) Po Valley, the (iv) western coast of France, and the (v) Nile delta^37^.

These regions are experiencing an increased vulnerability to ESLR due to a confluence of environmental and anthropogenic factors. In the North Sea region and United Kingdom, enhanced storm surges frequently exacerbate sea level rise, compounding the risks to coastal infrastructure and habitats. On the contrary, in the Po Valley and the Nile Delta^37^, subsidence due to natural and human-induced processes has resulted in significantly lower land elevations relative to sea level, heightening their vulnerability to flooding. Additionally, these areas suffer from varying degrees of inadequate coastal management, which fails to mitigate the effects of rising sea levels effectively. These factors combine to increase the frequency and severity of flooding^38^, posing substantial risks to ecological systems, economic stability, and human populations in these regions^33,36,39^.

Considering the five climate change scenarios previously mentioned, we observe that the area under threat of an ESLR event increases with the severity of the climate projection and the time frame considered. Thus—as reasonably expectable—the RCP8.5 scenario is much more severe than the RCP4.5, and similarly^30^, projections for 2100 are worse than those for 2050 and the Baseline scenario^29^. In the Baseline scenario, the estimated total area under the threat of ESLR along the coasts of Europe and North Africa ranges from 44,014 to 49,849 km^2^, with a median of 46,614 km^2^. These values remain relatively stable, with minor increases of 8% and 11% for the RCP4.5 and RCP8.5 scenarios, respectively, averaged across the three risk levels in the 2050 projection timeframe. However, more significant increases are observed in the projections for 2100. Specifically, the exposed area increases by 18% from the Baseline for the RCP4.5 scenario and by 35% for the RCP8.5 scenario 38. Although the Baseline scenario is preferable, we note a minor increase in 2050 without significant differences between the two RCP scenarios across the three risk levels. On the other hand, in the 2100 timeframe, we observe the most notable differences between the RCP8.5 and RCP4.5 scenarios, especially for the most severe events that are unlikely but can have the most destructive effects, leading to an increase of 24% in areas of low vulnerability. Given the escalation of these risks, it is imperative to integrate robust, forward-thinking coastal management strategies that incorporate both mitigation and adaptation measures tailored to these vulnerabilities, ensuring the resilience of affected communities against impending SLR, and associated extreme events^8^.

### From land cover to crop losses

Once the areas affected by ESLR have been estimated, further analysis to explore the economic and social impacts were performed. To this end, the ESLR areas are overlaid with Corine Global Land Cover^40^, and intersections are computed.

**Figure 2:**
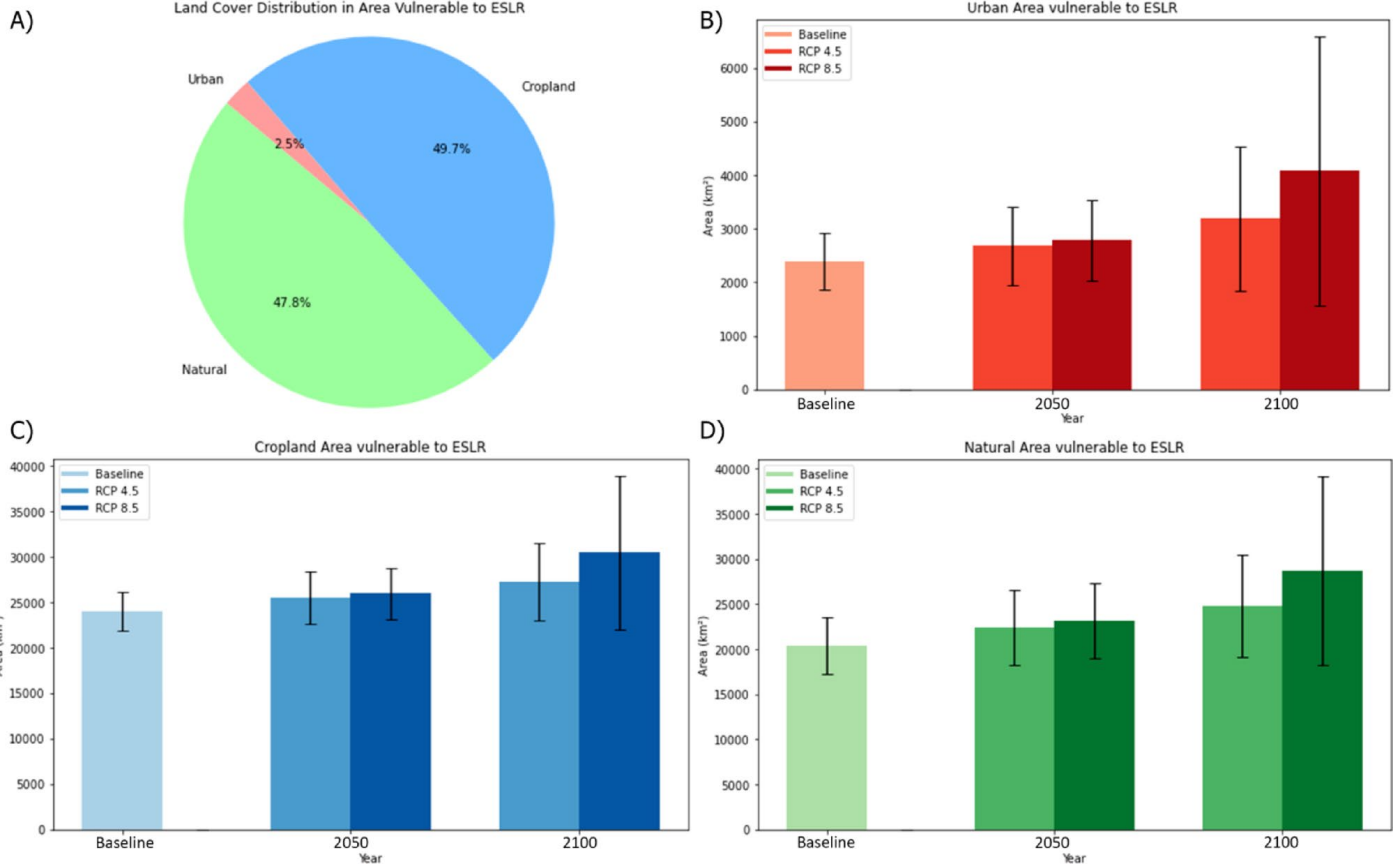
Pie graph showing the distribution of Land-Cover (3 classes) of the Impacted Areas (**A**). Bar graph showing the differential among the different scenarios, timeframes and vulnerability levels (expressed through the confidence intervals) for Urban (**B**), Cropland (**C**) and Natural (**D**) classes.

**Figure 3:**
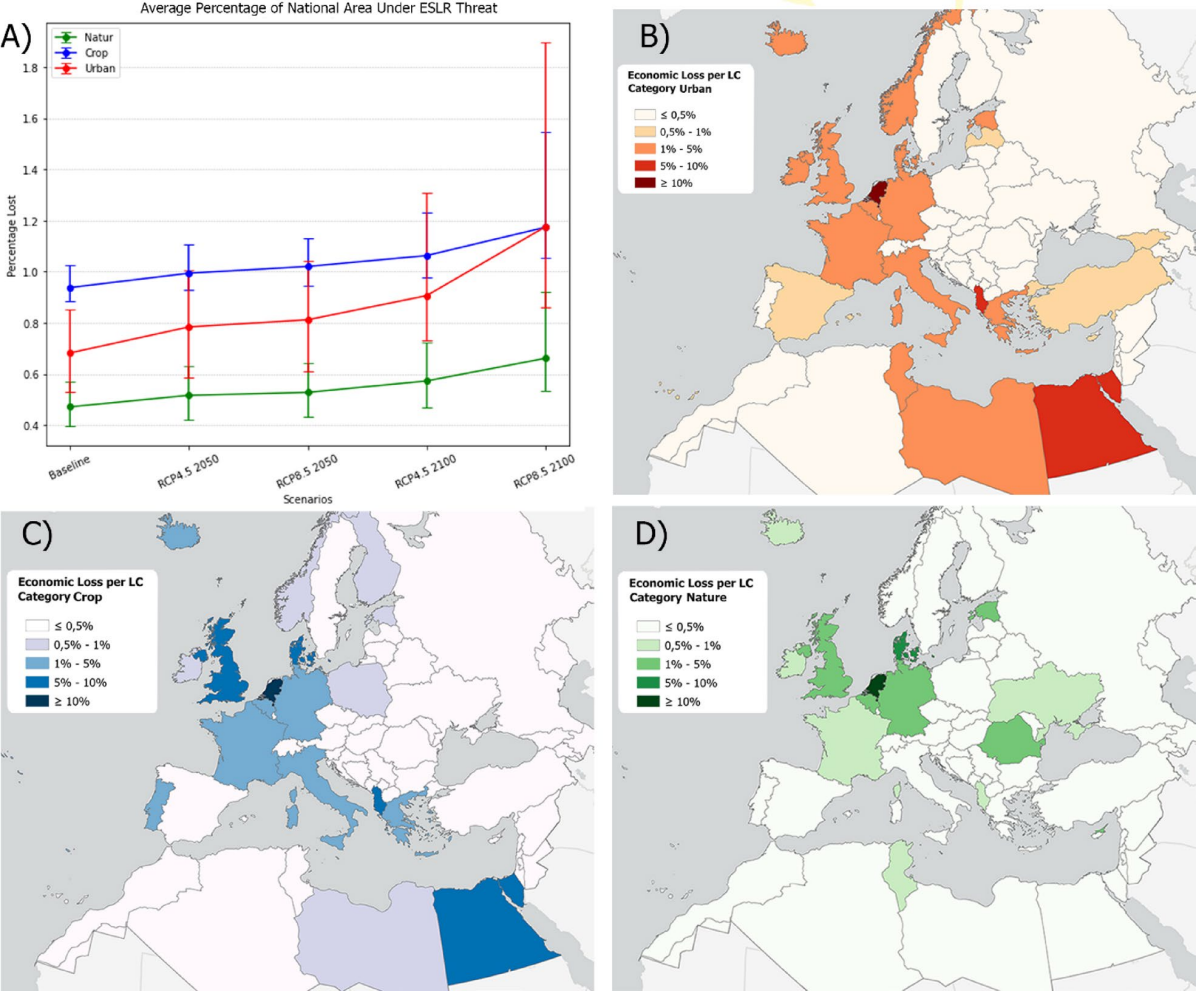
Plot of average percentage of national area under ESLR threat for 3 land cover classes (**A**). Map showing average percentage of area loss for built up (BA), cropland (CB) and natural land (DC) cover classes. Map created by the authors using QGIS (3.40, url: https://qgis.org/)) and Python (3.15).

In Fig. 2.A, we observe a significant prevalence of vulnerable cropland (Fig. 2.A and 2.C), which accounts for 49.7% of the total, in comparison to natural (47.8%) (Fig. 2.A and 2.D) and urban (2.5%) areas (Fig. 2.A and 2.B). Different patterns of changes in LC of affected area are also shown in Fig. 3.A. Cropland exhibits the least variability across different scenarios (Fig. 3.A and 3.C). This pattern can be explained by the spatial distribution of agricultural land, which is typically located in low-lying coastal areas close to river mouths and estuaries—regions historically favoured for their fertile soils, easy irrigation access, and transport connectivity^41^. Consequently, croplands are exposed even to moderate ESLR events, highlighting their persistent vulnerability. In contrast, urban areas exhibit a higher vulnerability to extraordinary events^24^ (Fig. 3.B)—which, although less likely, can have a more severe impact^42^—a different vulnerability profile: while their absolute exposure is lower, their sensitivity to catastrophic ESLR events is significantly higher. This reflects long-standing strategic urban planning practices that tend to locate cities slightly inland or to protect them via engineered defences such as levees, seawalls, and storm surge barriers^43,44^. This observation is consistent with strategic urban planning perspectives that position urban areas away from the coast or in locations protected by natural or artificial barriers^45^. Although cities are better shielded under moderate sea-level conditions, they remain critically exposed to extreme scenarios that can overwhelm both natural and artificial protections, leading to substantial damage to infrastructure and heightened risks to human lives^46^. Thus, our results underscore two distinct but complementary vulnerabilities: (i) cropland faces chronic exposure under a wide range of ESLR scenarios, posing systemic risks to food production; whereas (ii) urban areas face acute exposure during rare, extreme events, with potentially devastating social and economic consequences. The heightened vulnerability of cropland to ESLR poses a serious threat to European food security, due to the potential loss of agricultural production resulting from the flooding of high-value coastal farmland^42,44^ (Fig. 3.C). Many of Europe’s fertile regions, such as the river deltas in Italy, the Netherlands, and Egypt, are located near seacoasts. As ESLR continues to intensify with climate change, the potential for reduced agricultural output and compromised food supply chains could lead to increased food insecurity across the continent, highlighting the urgent need for resilient agricultural practices and enhanced coastal defences^45^.

### Assessing the agricultural impact of ESLR on coastal lands

Delving deeper into the economic analysis, we quantified the impacts of an ESLR event on agricultural production across various climate change scenarios. Utilising the GAEZ 2015 dataset^48^, which provides productivity data for 26 crops with a resolution of approximately 8.5 km^2^, we estimated the potential loss in agricultural production that could result from such an event^49^. By overlaying ESLR projections onto the GAEZ dataset, we calculated the percentage of each cell vulnerable to ESLR.

**Figure 4:**
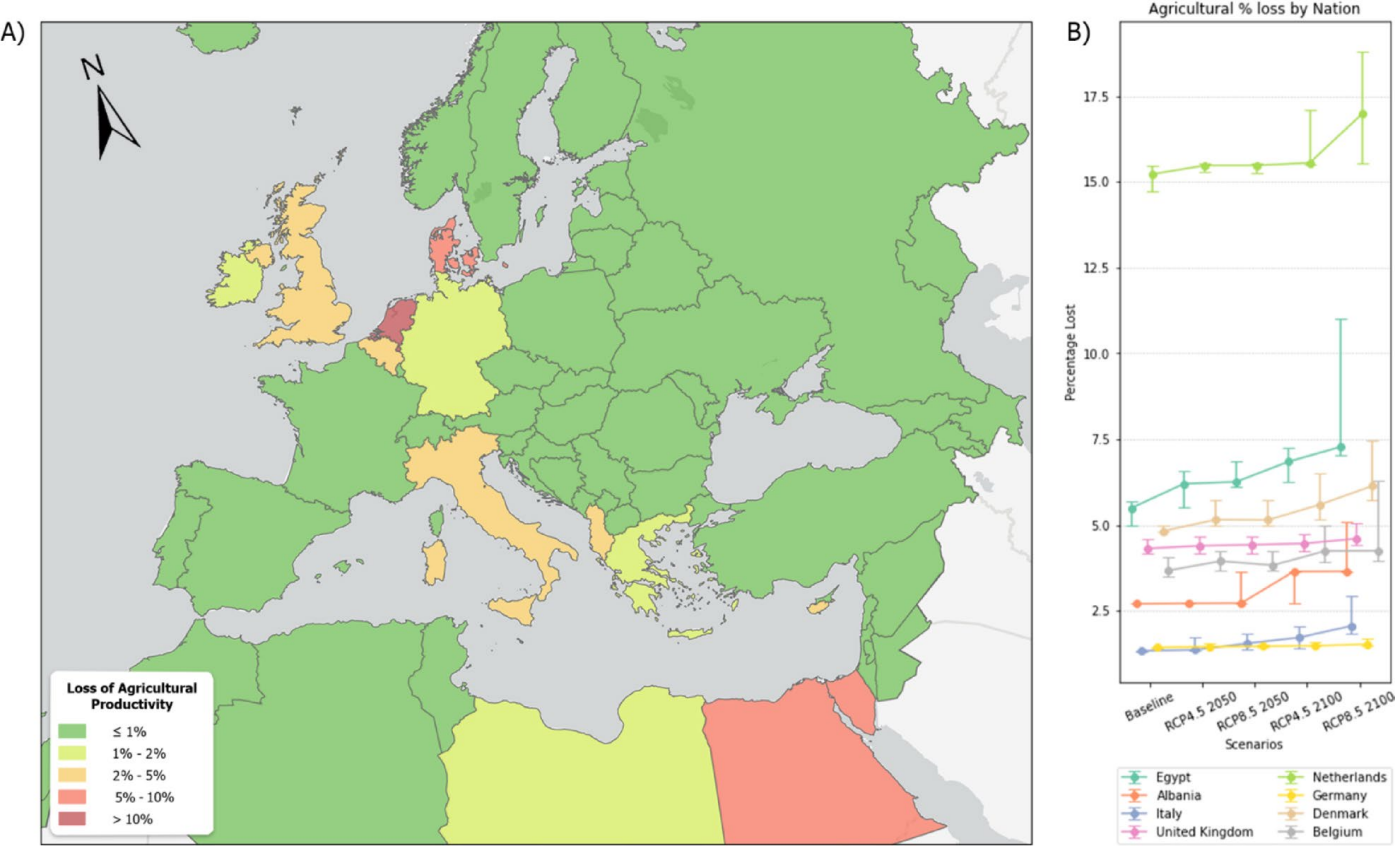
Average Percentage Loss of Agricultural Productivity on the total for each Nation (**A**). Line Graph showing the eight most vulnerable nations and their percentage across each scenario, timeframe and vulnerability level (reported as error bar) (**B**). Map created by the authors using QGIS (3.40, url: https://qgis.org/)) and Python (3.15).

We assumed no production in the year of the event and a uniform crop distribution within each cell, which enabled us to estimate the overall loss of agricultural productivity. The accompanying graph illustrates the average loss of agricultural productivity for each nation (Fig. 4.A). Notably, the Netherlands exhibits the highest percentage loss, ranging from % under the Baseline Scenario to 18.81% in the worst-case scenario. Other countries like Libya, Portugal, Italy, France, Germany, and Albania show losses ranging between 1 and 10%^50^.

## Discussion

Our projections suggest that the economic impact of ESLR on agriculture could be severe, particularly in regions where agricultural lands are near the coast. Specifically, the situation in Egypt illustrates how even modest exposure to ESLR can lead to disastrous impacts, given the region’s morphology and land use. The Nile delta, a densely populated and highly modified area, concentrates nearly all the country’s agricultural and other productive activities. An extreme ESLR event in this region would have catastrophic consequences, as reflected in our analysis. To estimate the economic impact of the phenomenon, the productivity data for each cell (measured in 1000 tonnes) is multiplied by the price per tonne as recorded by FAOSTAT for each nation^51^. This analysis yields intriguing results, revealing nations that may not appear highly impacted at first glance. Although Egypt remains the foremost in terms of total agricultural losses, The Netherlands, Turkey, France, and Germany also emerge as significantly affected, alongside the United Kingdom and Belgium, primarily due to the specific crops likely to be impacted by ESLR. The Netherlands due to its particular geomorphology is significantly affected despite the mitigation strategies adopted. These strategies include the construction of sea walls and dikes to protect, primarily the population and the major infrastructures, by ESLR events.

**Figure 5:**
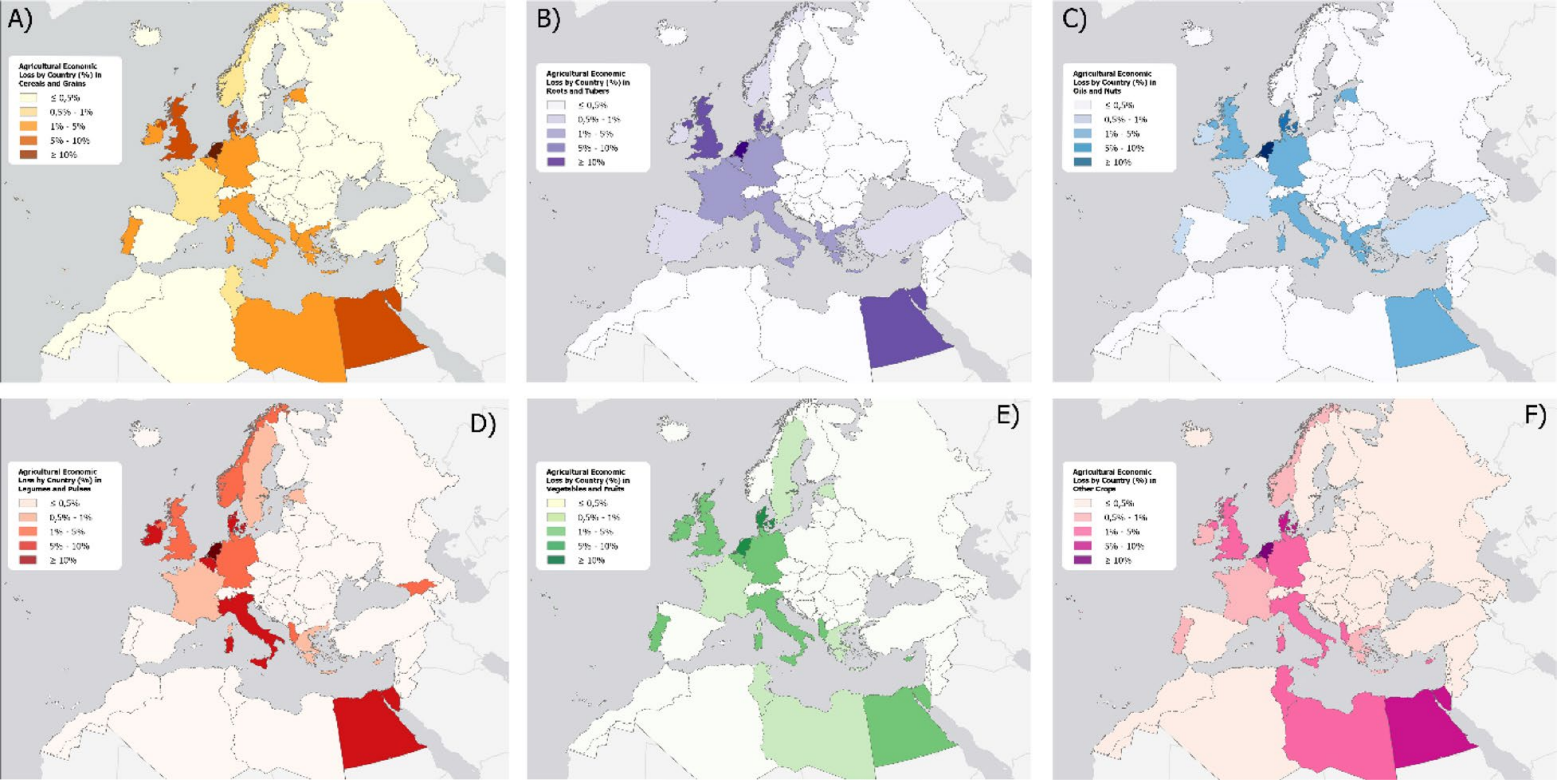
Average Percentage of Agricultural Economic loss on the total by nation for 6 categories of crops: Cereals and Grains (**A**), Roots and Tubers (**B**), Oils and Nuts (**C**), Legumes and Pulses (**D**), Vegetables and Fruits (**E**) and other crops (**F**). Map created by the authors using QGIS (3.40, url: https://qgis.org/)) and Python (3.15).

Turkey presents a notable case^52^, with certain areas showing vulnerability to ESLR, such as the district of Bafra. Situated in a low-lying region near the Black Sea coast and traversed by the Kızılırmak River, Bafra is minimally affected by storm surges from the Black Sea^51^. However, projections indicate that some areas are still at risk of flooding. Given that these lands are among Turkey’s most fertile and are utilised for growing high-quality tobacco, the economic repercussions could be substantial. When considering the total value of production vulnerable to ESLR across all crops and nations for the next 100 years^53^ the total amount spread from $800 million in the best-case scenario up to $1.5 billion in the worst-case scenario (Fig. 5).

These findings underscore the urgent need for proactive measures to safeguard agricultural productivity in the face of increasing risks from Extreme Sea Level Rise^11^. The experience of the Netherlands illustrates that it requires 25 to 50 years to get agreement and implement large scale coastal protection schemes^12^. The disparities in potential loss across different nations highlight the importance of region-specific strategies that consider local agricultural practices^54^, crop types, and the geographic vulnerabilities of each area. For nations like the Netherlands, where significant losses are projected even in less severe scenarios, the improvement of robust coastal defences and the development of salt-tolerant crop varieties could mitigate some of the negative impacts^51^. Additionally, enhancing early warning systems and improving regional planning can help to reduce the economic burden on nations with high-risk zones like the Nile delta and Bafra. Furthermore, recent research revealed that certain species known for biofuel potential cannot solely enable highly adaptive mechanisms to salinity stress (very high on land affected by ESLR), but even thrive in saline soils (i.e. *Schrenkiella parvula* of the Brassicaceae family)^55^, hence being an indication that agricultural strategies aiming at adapting to climate change adaptation may stimulate to reconsider current agricultural pattern, favouring biofuel crops on saline soils, so to reserve non-saline arable lands for cash crops.

Given the economic stakes involved, international cooperation and funding for research into resilient agricultural practices^15^ and climate adaptation technologies will be critical^46^. These collaborative efforts should aim not only to prevent immediate losses but also to ensure the long-term sustainability^56^ of food production systems globally, protecting them against future ESLR events and other climate-related challenges^48^.

## Methods

### ESLR vulnerable area identification

In this study, we utilised the ESLR projections developed by Vousdoukas et al.^21^ to delineate areas vulnerable to Extreme Sea Level Rise (ESLR) events across Europe and North Africa. ESLR projections by Vousdoukas et al.^21^ were selected because they offer the most up-to-date and spatially comprehensive estimates, grounded in a robust climatological framework. Their dataset distinguishes between Relative Sea Level Rise and extreme events, while also providing unified ESLR metrics and 100-year return period values in meters—facilitating direct integration with elevation data and enabling consistent risk and economic loss assessments. A raster map with a resolution of 90 metres was produced to highlight these regions at risk. The spatial model developed incorporates the DeltaDTM^26^, a coastal elevation dataset with an approximate resolution of 30 metres (for Europe and North Africa), and the AHN DEM, a 0.5 DTM (0,5 metre spatial resolution) only for the Netherlands. Hence, a finer dataset is used for the Netherlands (AHN, 0.5 m resolution), where detailed elevation data and extensive protective infrastructure are available. This allows for a more accurate representation of human-made barriers such as dikes and sea walls. For other countries, we include major coastal protection elements using data from the Climate Technology Centre & Network (CTCN), which provides global information on coastal defenses. This is because the Netherlands, since the devastating North Sea flood of 1953, is the only country that has implemented extensive flood protection measures, allowing the country to effectively mitigate the impacts of sea level rise and extreme sea level events, to safeguard not only agricultural land, but also densely populated areas, socio-economic activities, and critical infrastructure.

Other countries like United Kingdom, Italy, Germany and United State have implemented a few sea barriers and dikes’ systems which are not comparable with the integrated Netherlands’ system. Nonetheless, to consider these elements also for other countries an original dataset with dikes and sea barriers based on Climate Technology Centre & Network^60^ was created and then merged with the DTM. In this way we ensure to consider within the analysis human made adaptation structures that massively reduce the impact of ESLR in coastal areas in which they are presents. Consequently, our assessment should be interpreted as reflecting a no-adaptation baseline for most countries, with limited exceptions where data permit. While this approach enables partial integration of large-scale structural measures, we acknowledge that localized or smaller-scale protections may not be fully captured due to the absence of harmonized and high-resolution data across the entire study area. Therefore, future efforts should prioritize the integration (which is easily implementable in our model) of more complete datasets on coastal protection to better represent adaptation scenarios at finer localized scale when these will become available. To this end, individual tiles in .tif format, were processed in Google Earth Engine using JavaScript scripts.

While the ESLR projections we employ already reflect dynamic interactions between tides, waves, and storm surges in open coastal waters, our modelling approach translates these projected sea levels into spatially explicit land impacts. To do so, we use a hydrological connectivity method that takes into account not only absolute elevation but also topographic flow paths and slope. This method improves upon simple “bathtub” models by preventing unrealistic inland spread of water over isolated basins and offers a practical compromise between physical realism and scalability. While it does not simulate the full complexity of coastal hydrodynamics or wave run-up, it provides a reliable and repeatable framework for assessing flood exposure across large geographic areas and under different future ESLR scenarios.

To integrate ESLR scenarios with a 100-year return period provided by the Joint Research Centre (JRC) in .csv format, we converted these files into .shp point format. Spatially distributing the point data required a lattice of Thiessen polygons, constructed through Voronoi diagram. This methodological approach ensures that each area within the model is associated with the nearest coastal projection point, facilitating a continuous spatial analysis^59^. The Thiessen polygons were generated using ArcGIS Pro and subsequently rasterized and uploaded in Google Earth Engine (GEE) for further processing.

The rationale to identify the ESLR vulnerable areas is^1^:

$$\begin{aligned} & R(x,y) = \left\{ {\begin{array}{*{20}l} 0 \hfill & {{\mathrm{if}}\;(x,y) \in L\;{\mathrm{and}}\;DEM(x,y) \le ESLR(x,y)} \hfill \\ {1 - \exp \left( { - \frac{{\min \sum\nolimits_{i \in \epsilon } {(1 + Slope(DEM(x,y)))*d(x,y)} }}{\sigma }} \right)} \hfill & {{\mathrm{if}}\;(x,y) \in L\;{\mathrm{and}}\;DEM(x,y)> ESLR(x,y)} \hfill \\ 0 \hfill & {{\mathrm{if}}\;(x,y) \in S} \hfill \\ \end{array} } \right. \\ & {\mathrm{where}} \\ & ESLR(x,y) = ESLR(p_{i} ) \\ & (x,y) \in V_{p} (p_{i} ) \\ & V_{p} (p_{i} ) = \{ q \in R^{2} :d(q,p_{i} ) \le d(q,p_{j} ),\forall j \ne i \\ \end{aligned}$$

Given L and S, the two sets representing points on the land and in the sea, respectively, and having defined the functions: $DEM\left( {x,y} \right)$, which identifies the elevation above sea level for each pair of coordinates, and $ESLR\left( {p_{i} } \right)$, which associates each point on the coast, $p_{i}$, with the relative value of the height of the extreme sea level event, we defined the risk function R function of $DEM\left( {x,y} \right)$ and $ESLR\left( {x,y} \right)$ the Voronoi transformation of $ESLR\left( {p_{i} } \right)$. This function identifies vulnerable areas, assigning them a value between 0 and 1, while non-vulnerable areas and open waters are considered Null. The risk function is defined as the inverse of the exponential of the cost distance function *R(x,y)*. The cost function is a function that quantifies the resistance or difficulty of the sea level event to propagate inland, depending on local topographic gradients, surface roughness, and the continuity of low-lying terrain. In other words, areas with low elevation and high connectivity to the coastline will have a lower cost and therefore higher risk values, while areas at higher altitudes or separated by significant topographic barriers will present higher costs and consequently lower risk scores. In this way distance from the coast, terrain morphology and exponential decay of the flood are while considered. To do so the GEE (an Arcpy Python library used for displaying map tiles and printing rich representations of Earth Engine) was used to compute the differences between the DEM and the ESLR projections. Cells yielding a positive difference were assigned a *null* value, indicating areas not affected by ESLR, whereas those holding negative values were given value equal to 1, eliciting areas likely to be impacted by ESLR. Then, these areas were used to spatially mask Slope raster layers derived using the TAGEE package in GEE. These raster strata were exported and processed in ArcGIS Pro. The previously defined risk function was implemented in the ESRI environment using the Distance Accumulation tool, where the slope layers served as the cost raster, the OpenStreetMap (OSM) coastline was set as the source of flooding while the original Sea Barriers shapefile was set as inundation barrier. This operation is critical, because it excludes false positive results, such as depressed areas below sea level, with no spatial connection with open waters whatsoever, and/or situated hundreds of kilometres from the coast. Lastly, an inverse exponential function was applied according to Boettle et al. (2016) to model inundation expansion^58^.This procedure was run for all the possible permutations of: climate change scenarios, timeframes, and vulnerability levels. Thus, the procedure resulted in 15 single band tif rasters. Each raster has pixels with values ranging from 0 to 100, eliciting the area of each pixel under the threat of an ESLR event. In this way, we produce spatially explicit maps showing the spatial extent of an ESLR event happening with a marginal incremental probability of 1% each year.

### Land cover assessment

Vulnerability mapping offers significant insights for environmental and socio-economic analysis, particularly through the exploration of land cover within areas susceptible to ESLR. Our initial approach involves a detailed examination of land cover characteristics^47^. We employed a methodology that overlays the projected geographic extent of future ESLR projections onto current land-use maps, enabling the assessment of potential impacts across different land-use categories. Specifically, we utilised the Corine Global Land Cover^40^, which we reprojected into EPSG:3035, featuring a resolution of approximately 100 metres^23^. To streamline the analysis, we consolidated the 20 classes representing natural elements—excluding those designated as ‘Built Up’ (code 40) and ‘Croplands’ (code 50)—into a single ‘Natural’ class, resulting in three primary categories: Built Up, Cropland, and Natural^10^.

Subsequently, assuming land-use patterns and associated values would remain constant in the future, the reclassified Corine layer is overlaid with the 15 distinct ESLR projection scenarios. This integration assigns land cover values to the corresponding ESLR layers, facilitating the classification of each pixel based on its vulnerability to sea level rise. Then the Arcpy ‘Tabulate Area’ function is used to calculate the area covered by the pixels within each class. The results of this spatial analysis were meticulously compiled and stored in a tabular format^29^. This refined approach not only enhances our understanding of the potential spatial distribution and intensity of ESLR impacts but also supports robust decision-making for mitigation and adaptation strategies in vulnerable coastal regions highlighting the importance of adopting it also in the agricultural sector.

### Agricultural production losses and economic impacts

Given that 49.7% of the land affected by extreme sea level rise (ESLR) is agricultural, we proceeded to assess potential land-capability and crop value losses due to such events. For this analysis, we utilised the Global Agro-Ecological Zones (GAEZ) 2015 dataset. This dataset offers global, gridded data (at 5-arcminute resolution) on irrigated and rainfed crop areas, production, and yield across 26 different crops and crop categories, based on national statistics. Firstly, the .tif files from GAEZ 2015 were reprojected into the ETRS 1989 LAEA (EPSG: 3035) coordinate system. Using the Python GDAL library, we quantified the ESLR vulnerable cells (~30m) within each GAEZ cell (~8.6km). Then we calculate the complement of the percentage for each GAEZ cell vulnerable to ESLR. This results in a raster ranging from 0 to 1 where cells with value 1 indicate no ESLR impact, whereas those with value 0 would be completely affected by an ESLR event. This derived raster was then multiplied by the GAEZ productivity raster to estimate agricultural productivity losses for each of the 15 climate change scenarios and across the 26 crops^47^. This calculation assumes a uniform distribution of cultivated areas within each cell and a constant crop mix over time. These assumptions enabled us to estimate, with reasonable accuracy, the total agricultural production for each nation and the corresponding losses attributable to ESLR events^43^.

**Figure 6:**
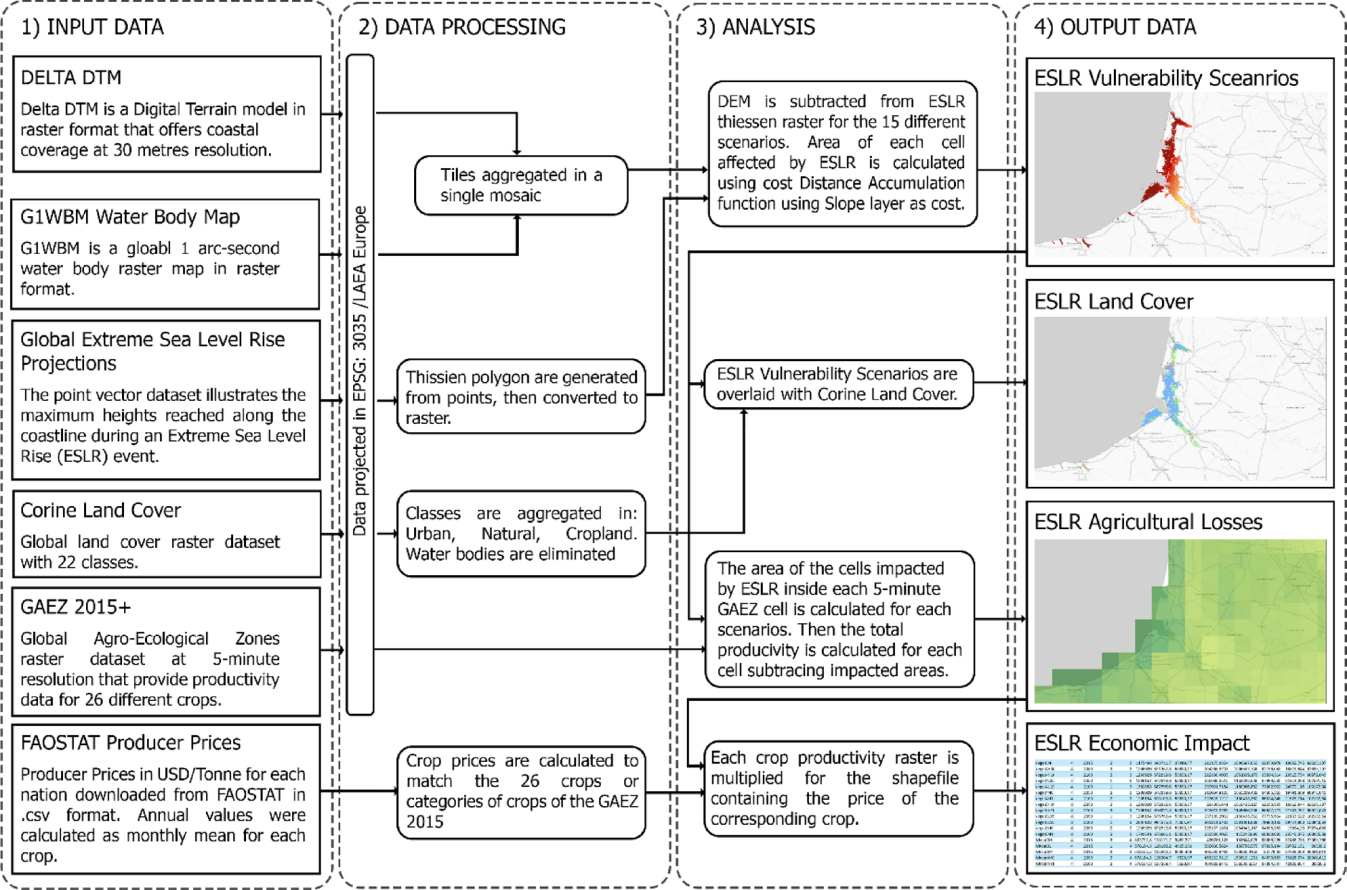
Methodological procedure followed to achieve the 4 products elaborated in this work.

To estimate the economic impact of an ESLR event, we multiplied the raster of the agricultural productivity (in 1000 tonnes per pixel) for each of the 15 scenarios by the producer price per tonne of each crop by nation. The price of the 26 crops—or group of crops—are downloaded by FAOSTAT, and the crop categories are created following the GAEZ 2015 metadata. Then, zonal statistics are calculated to aggregate the data at the national level (NUTS-0). These crops are also grouped in 6 major classes according to FAO classification. This enables a better understanding of agricultural macro trends and sector specific elaborations (Fig. 6). Other descriptive statistics are calculated to complete the analysis and the results are displayed in maps. Interesting results come out in poorer regions that with good coastal land management could lower the risks linked to an ESLR event^18^.

### Future perspectives and key limitations

This study has crucially delineated the regions vulnerable to Extreme Sea Level Rise (ESLR), fostering a deeper understanding of the potential risks and enabling the development of targeted strategies for coastal land management and mitigation^12^. Through meticulous analysis using cutting-edge geographic and economic models, our findings highlight the profound impacts of ESLR on coastal regions, with a particular focus on agricultural lands, which encompass 58% of the areas at risk. Our research underscores the critical need for efficient mitigation strategies to reduce exposure and enhance resilience, particularly in agricultural zones that sustain significant economic and social value. The integration of ESLR projections with the GAEZ dataset has provided a novel perspective on the potential economic impacts, illustrating not only the immediate threats but also the long-term challenges to food security and regional economic stability. These impacts are especially pronounced in regions like the Nile Delta, where even modest ESLR events can have catastrophic consequences due to the area’s dense population and agricultural reliance^13^.

Furthermore, the methodological advancements in this study offer a robust framework for future research and policymaking. By combining high-resolution DTM, sea barriers data with detailed crop and economic data, we have enabled more precise mapping of ESLR vulnerability and its economic implications. Additionally, the study uses a comprehensive methodological approach which takes into consideration morphological aspects and flood hydrology that was never applied at large scale. This approach not only enhances our understanding of the spatial distribution of risks but also supports the formulation of region-specific adaptation strategies, which are essential in the face of increasing ESLR incidents. However, the study is not without limitations^18^. The resolution of the global DTM employed, while among the highest currently available, may inadequately represent smaller-scale geographic features that influence vulnerability assessments, particularly anthropogenic structures such as embankments, dykes, and water gates designed to prevent seawater intrusion. Using 0.5 meters DTM for the Netherlands and creating a dataset with human made features we believe to have addressed this issue effectively; yet, extremely localized case studies aiming at mimicking the proposed methodology may further benefit from the adoption of place-based higher resolution DTM and infrastructure data.

Another important methodological point concerns the flood modelling approach adopted in our study. We recognize that our topographic model does not simulate complex hydrodynamic processes such as storm surge interactions, wave run-up, or tidal amplification. However, the ESLR projections we used as inputs—particularly those from Vousdoukas et al. (2018)—already account for these dynamic drivers, offering a robust estimation of the expected extreme sea levels at the coastline under different climate scenarios. Our model builds on these projections by translating them into inland exposure patterns, using a cost-distance approach that incorporates elevation, slope, and proximity to the shoreline. While not hydrodynamic, this method allows for the realistic approximation of overland flood extent at the continental scale, and strikes a balance between accuracy and computational feasibility. It offers a substantial improvement over static “bathtub” models, while remaining applicable to large spatial domains where high-resolution hydraulic simulations are not feasible. This modelling framework, while simplified, provides an effective and replicable approach for identifying exposure hotspots and supporting regional-scale adaptation planning.

Furthermore, we acknowledge that one potential limitation of our analysis is the assumption of static crop distribution, which holds the spatial allocation and composition of agricultural land constant over time. As such, our economic loss estimates do not account for potential future adaptations in cropping patterns or land-use changes that may occur in response to climate pressures, market shifts, or technological developments. While we fully agree that these dynamics are important and merit exploration in future work, their inclusion would require spatially explicit projections that are not currently available at the resolution and scope of our study. But more importantly, a key objective of our work is to provide a clear and tangible risk assessment for policy makers and local stakeholders based on the agricultural systems that exist today. We believe that referencing current crop distributions allows for a more immediate and relatable understanding of what is at stake under (ESLR) scenarios. In contrast, presenting outcomes based on hypothetical future agricultural systems—while scientifically valid—would risk obscuring the direct implications for today’s communities, food systems, and regional economies; not to mention that these forward-looking scenarios also carry considerable uncertainty and depend on assumptions that would amplify the aggregate uncertainty of the results produced. For these reasons, we have chosen to ground our analysis in present-day agricultural configurations, providing a conservative yet policy-relevant estimate of potential ESLR impacts. This choice supports the study’s core aim: to raise awareness of the risks posed to current agricultural landscapes and to inform timely and targeted adaptation strategies.

Considering these findings and limitations, it is imperative for future research to continue in refining the spatial and economic models of ESLR impact^19^. Enhanced modelling precision, coupled with dynamic economic and agricultural data, will be crucial in developing more effective adaptation and mitigation strategies. A higher resolution DTM that would also consider artificial dykes and sea walls would return more faithful and current predictions (although, to the best of our knowledge, this is not available for the scale of analysis implemented in this work). These strategies must be tailored not only to the physical and economic landscapes but also to the socio-cultural contexts of the affected regions, ensuring that the most vulnerable communities are equipped to withstand the challenges posed by climate change. While this study does not conduct a full cost–benefit analysis of adaptation options, we caution that narrowly framing such comparisons around agricultural value may be misleading. Coastal areas often concentrate multiple vital assets beyond cropland, including densely populated settlements, infrastructure, and cultural heritage sites. Therefore, protective measures should be evaluated not only for their economic return, but also for their societal necessity. Nevertheless, by reporting national-level cropland exposure rates, this study offers a preliminary yet policy-relevant tool for identifying regions where adaptation planning may be most urgently required. Therefore, our investigation, by providing a comprehensive assessment of the risks and a detailed blueprint for mitigation, contributes a critical piece to the puzzle of climate resilience^58^. It calls for an integrated approach that combines scientific inquiry with practical policymaking^59^, leveraging international cooperation and innovation to safeguard our most vulnerable coastal regions against the impending challenges of Extreme Sea Level Rise^21,61^.

## Supplementary Information

Below is the link to the electronic supplementary material.

Supplementary Material 1

## Data Availability

Raster files of the mapped vulnerability to ESLR according to the climate change scenarios considered, and shapefiles containing vulnerable areas to ESLR and the related agricultural losses (both in square meters and percentage) are accessible and available for download at the following link: https://data.mendeley.com/datasets/932rzcschf/1 . Additionally, result maps can be visualized and queried through the online platform GEE at the following link https://gee-ecogeo.projects.earthengine.app/view/elsr-vulnerability. Further information and dataset can be provided by contacting the corresponding author at federico.martellozzo@unifi.it.
