# Supplementary material for: Assessing extreme sea level rise impacts on coastal agriculture in Europe and North Africa
Source: Sci Rep. 2025 Dec 20;16:1939. doi: 10.1038/s41598-025-31630-w (PMC12804789; doi:10.1038/s41598-025-31630-w)
Supplement: Supplementary file 1 — Supplementary Material 1 [file 41598_2025_31630_MOESM1_ESM.docx]

**Supplementary Materials**

Rising Tides, Sinking Crops. Assessing Extreme Sea Level Rise Impacts on Coastal Agriculture in Europe and North Africa

Federico Martellozzo ^i,^*, Matteo Dalle Vaglie ^i^, Filippo Randelli ^i^, Carolina Falaguasta ^i^, Pim van Tongeren ^ii^, Katarzyna Negacz ^ii^ , Bas Bruning ^iii^ , Pier Vellinga ^ii^

* Corresponding author, federico.martellozzo@unifi.it

^i^ University of Florence, Dept. of Economics and Management (DISEI).

^ii^ Vrije Universiteit Amsterdam, The Institute for Environmental Studies (IVM)

^iii^ The Salt Doctors

**SM1) Brief description of ESLR input projections used (i.e. from Vousdoukas et al. 2018)**

**Projections developed by Vousdoukas et al. (2018) were selected over alternative datasets based on several methodological and practical considerations. First, to the best of our knowledge, these projections represent the most recent and comprehensive Extreme Sea Level Rise (ESLR) estimates available, incorporating both global coverage and regional specificity. Second, the study is grounded in a robust climatological framework that integrates Representative Concentration Pathways (RCPs) with state-of-the-art hydrodynamic and statistical modeling techniques, ensuring both scientific reliability and internal consistency. Third, an important strength of the dataset lies in its clear decomposition of ESLR into its principal components—Relative Sea Level Rise (RSLR) and extreme coastal events—while also providing unified ESLR metrics. This allows for a more nuanced understanding of the drivers of future coastal flooding. Fourth, the dataset offers complete spatial coverage of our study area, which is essential for coherent cross-regional comparisons. Finally, the provision of ESLR heights associated with a 100-year return period, expressed in absolute elevation values (meters), enables straightforward integration with elevation models and facilitates the estimation of physical and economic impacts under the assumption of a 1% annual probability of occurrence**.

**SM2) ESLR Scenario point dataset.** Map created by the authors using QGIS (3.40, url: https://qgis.org/)) and Python (3.15).


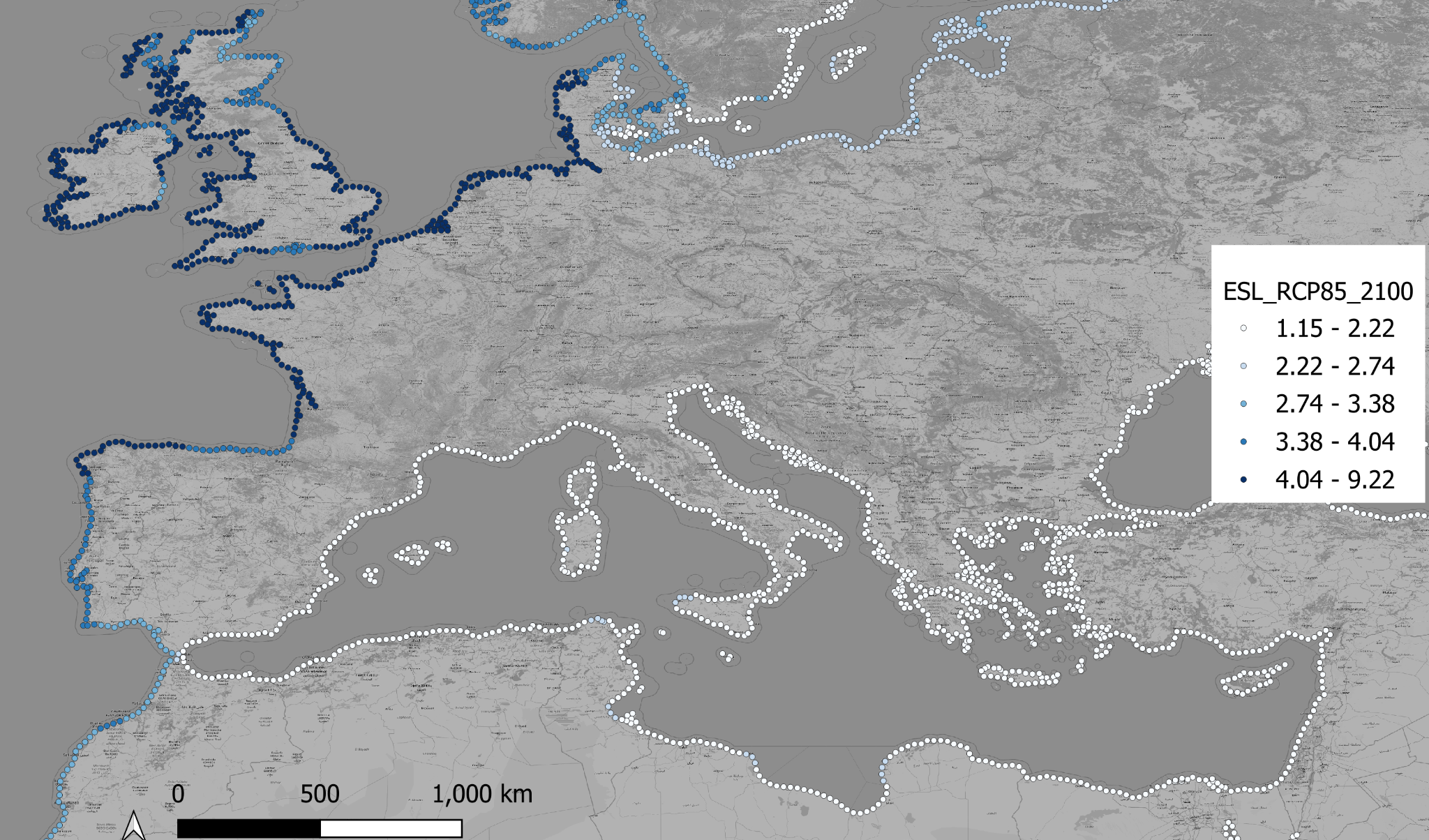


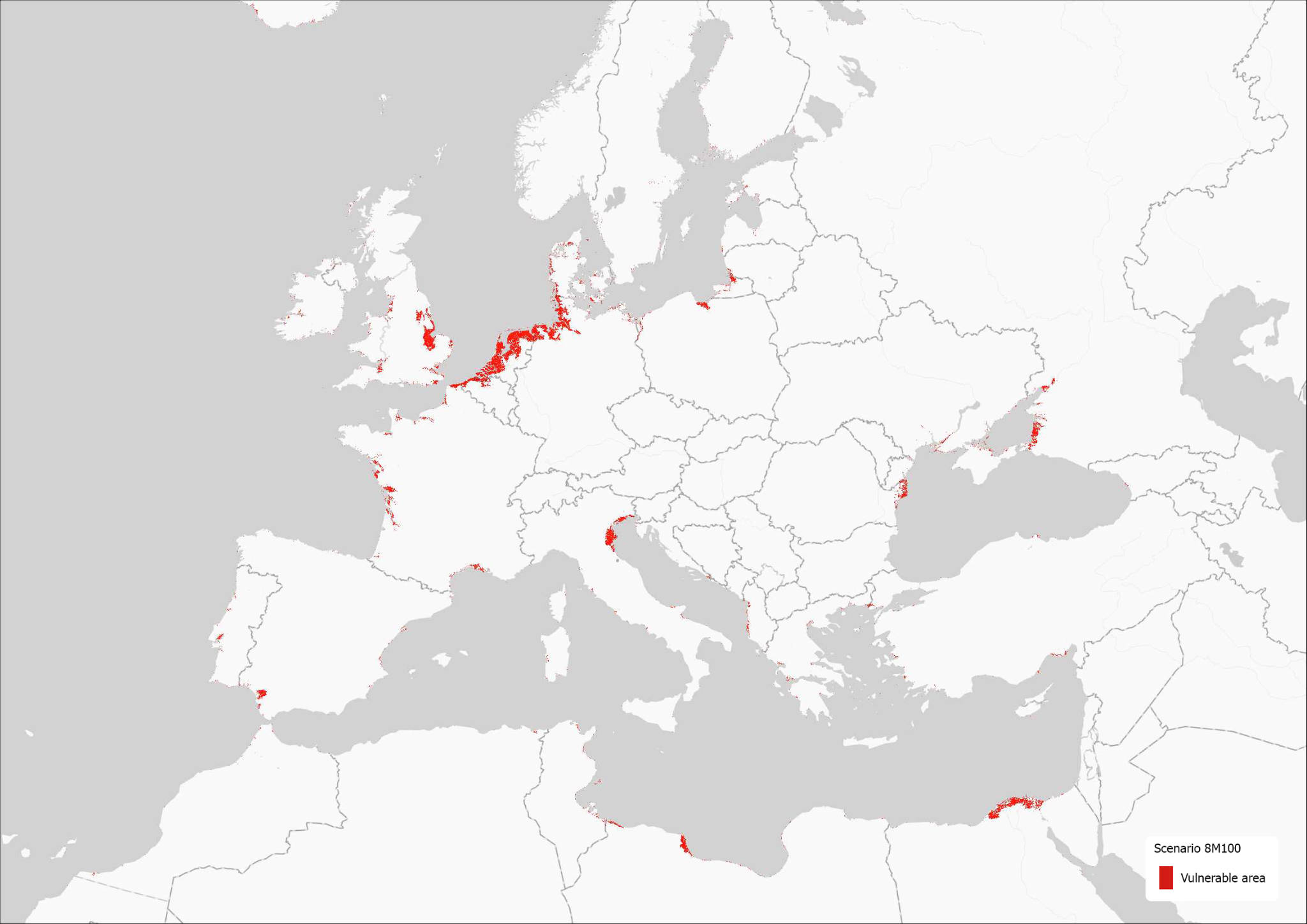

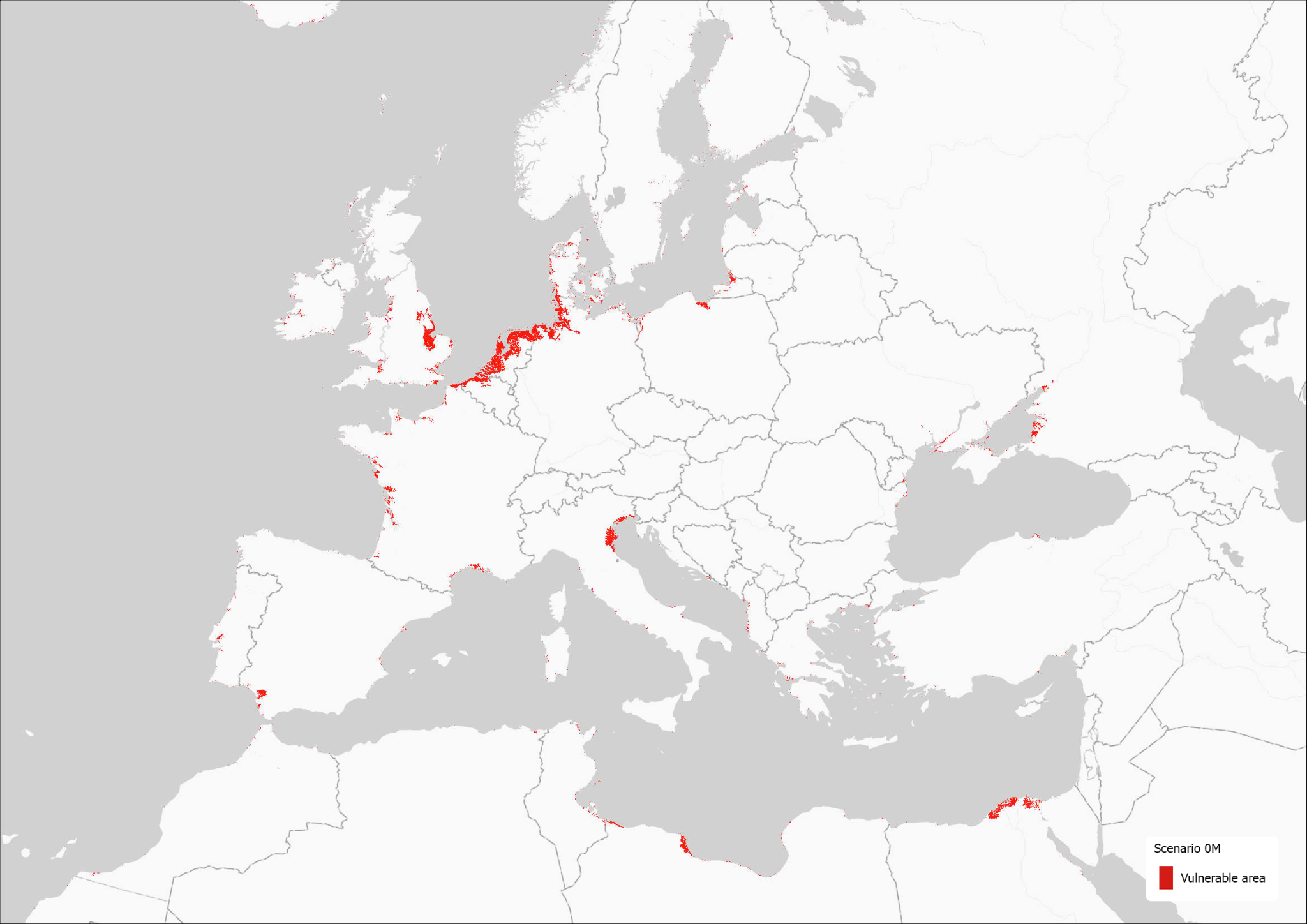
**SM3) ‘Business as Usual’ and ‘RCP8.5 2100’ scenarios medium vulnerability hitted area side by side comparison.** Map created by the authors using QGIS (3.40, url: https://qgis.org/)) and Python (3.15).


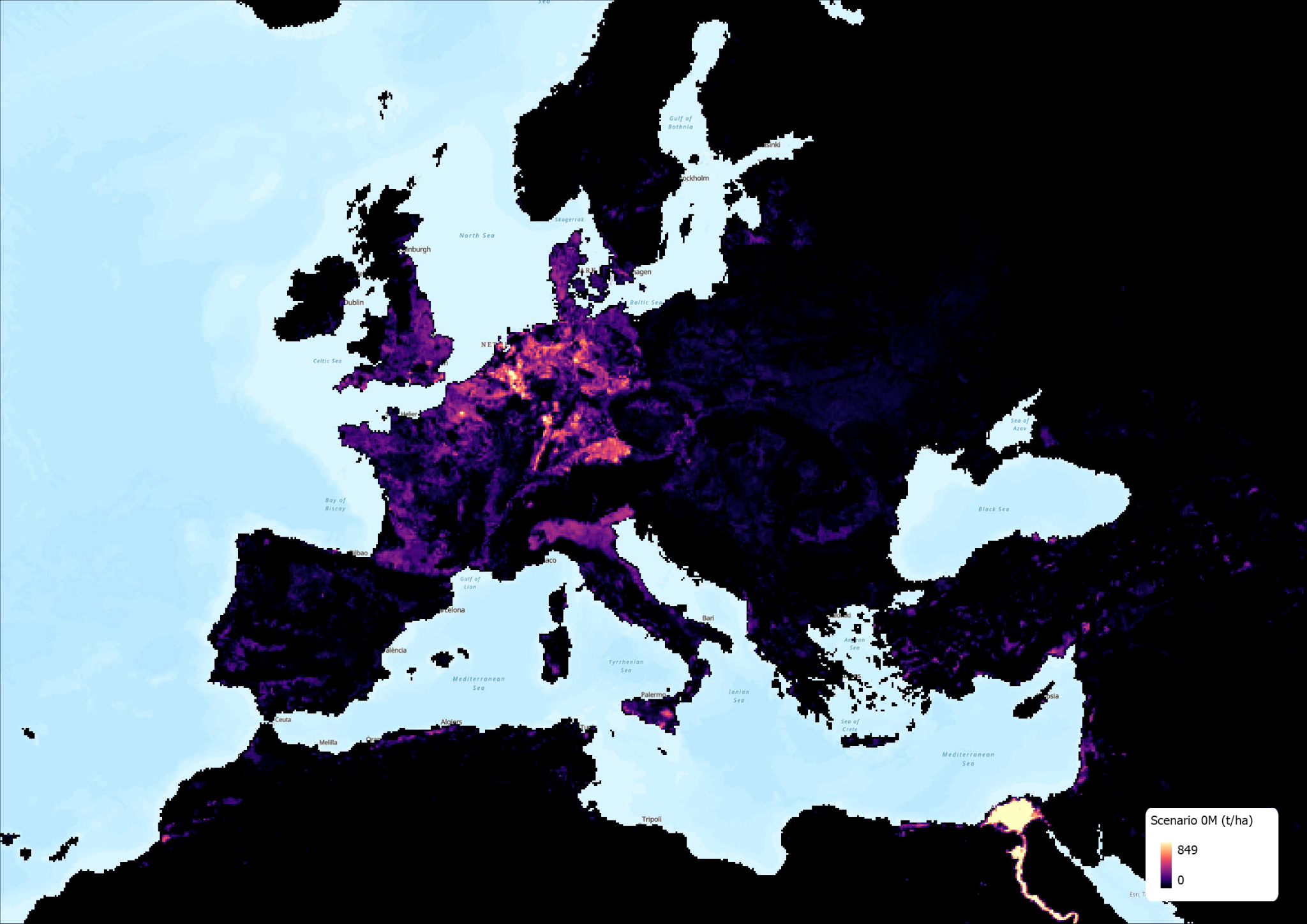
**SM4) ‘Business as Usual’ and ‘RCP8.5 2100’ scenarios medium vulnerability side by side comparison.** Map created by the authors using QGIS (3.40, url: https://qgis.org/)) and Python (3.15).


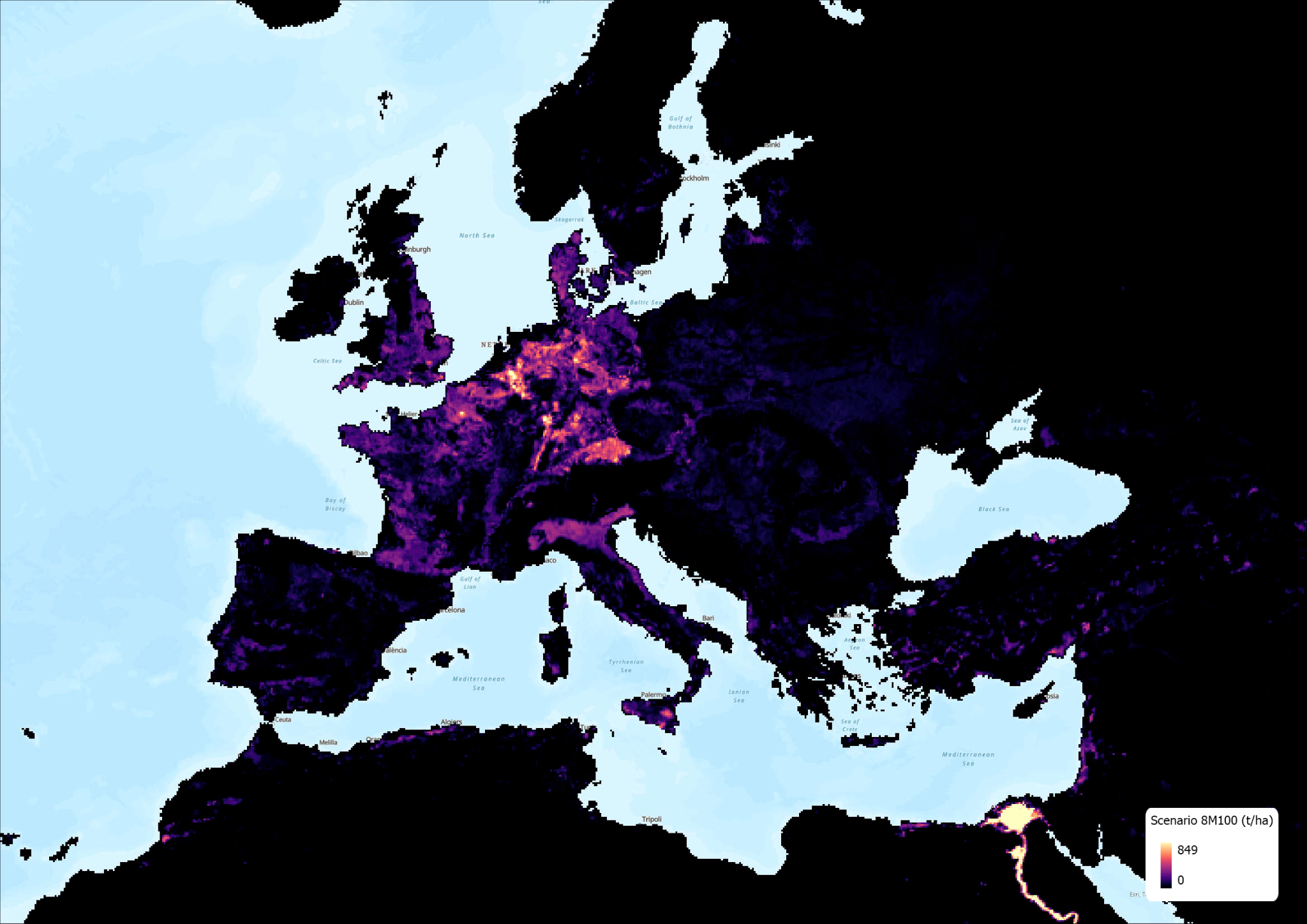


**SM5) Description of GAEZ crop dataset from FAO**

**In brief, our analysis is based on the Global Agro-Ecological Zones (GAEZ v4, 2015) dataset developed by FAO and IIASA, which provides global estimates of crop distribution, yield, and potential productivity at a spatial resolution of 5 arc-minutes (~10 km at the equator). The dataset includes crop-specific information on harvested area and potential yields under different management levels and climate scenarios.**

**We utilize the 26-crop classification provided in the GAEZ dataset, which aligns with the FAO’s standard crop classification system (FAO, 2010). This classification is essential for maintaining compatibility across global datasets and for ensuring consistency in quantifying production and loss estimates under ESLR scenarios. Our approach overlays areas exposed to ESLR with the GAEZ crop distribution maps, enabling us to estimate the expected production loss for each crop group under different flooding scenarios. The classification covers major cereals, pulses, oil crops, roots and tubers, industrial crops, and others, as summarized in the table below.**

**For a full reference to the FAO classification, please refer to:**

**FAO (2010). World Programme for the Census of Agriculture 2010: Appendix 3 - Classification of crops.**

**see: chrome-extension://efaidnbmnnnibpcajpcglclefindmkaj/**[**https://www.fao.org/fileadmin/templates/ess/documents/world_census_of_agriculture/appendix3_r7.pdf**](https://www.fao.org/fileadmin/templates/ess/documents/world_census_of_agriculture/appendix3_r7.pdf)

| **Crop Group** | **Examples** |
| --- | --- |
| **Wheat** | **Durum wheat, common wheat** |
| **Rice** | **Paddy rice** |
| **Maize** | **Grain maize** |
| **Barley** | **Barley** |
| **Millet** | **Pearl millet, finger millet** |
| **Sorghum** | **Sorghum** |
| **Pulses** | **Beans, peas, lentils** |
| **Soybean** | **Soybean** |
| **Groundnut** | **Peanuts** |
| **Sunflower** | **Sunflower seed** |
| **Rapeseed** | **Rapeseed** |
| **Oil palm** | **Oil palm** |
| **Sugarcane** | **Sugarcane** |
| **Sugar beet** | **Sugar beet** |
| **Potato** | **Potato** |
| **Cassava** | **Cassava** |
| **Sweet potato** | **Sweet potato** |
| **Banana/Plantain** | **Bananas, plantains** |
| **Cotton** | **Cotton** |
| **Coffee** | **Arabica, robusta** |
| **Cocoa** | **Cocoa beans** |
| **Tea** | **Tea leaves** |
| **Tobacco** | **Tobacco** |
| **Other cereals** | **Rye, oats** |
| **Other oil crops** | **Sesame, linseed** |
| **Other crops** | **Horticultural and minor crops** |

**SM6) Scenarios Name Convention Used**

The Scenarios are called for example: Banan4H050

- Where the first 5 digits represent the name of the Crop
- The 6^th^ digit tells us the RCP Scenario (0= Baseline, 4 = RCP4.5 and 8 = RCP8.5)
- The 7^th^ digit tell us the vulnerability level (L = 95^th^, M = 50^th^, H = 5^th^)
- The last 3 digits identify the year of the prevision 050 for 2050 and 100 for 2100

Crop Categories

- Cereals and Grains: 'Barle', 'Maize', 'Mille', 'Rice', 'Sorgh', 'Wheat', 'Other'
- Roots and Tubers: 'Groun', 'Potat', 'Yamsa',’Cassa’
- Oilseeds and Nuts: 'Olive', 'Rapes', 'Sunfl', 'Cotto', ‘Oilpa’
- Legumes and Pulses: 'Pulse', 'Soybe'
- Vegetables and Fruits: 'Veget', 'Banan', 'Crops'
- Commercial and Specialty Crops: 'Tobac', 'Stimu', 'Sbeet', 'Scane', 'Fodde'

SM7) Non Linearity of ESLR scenarios

**
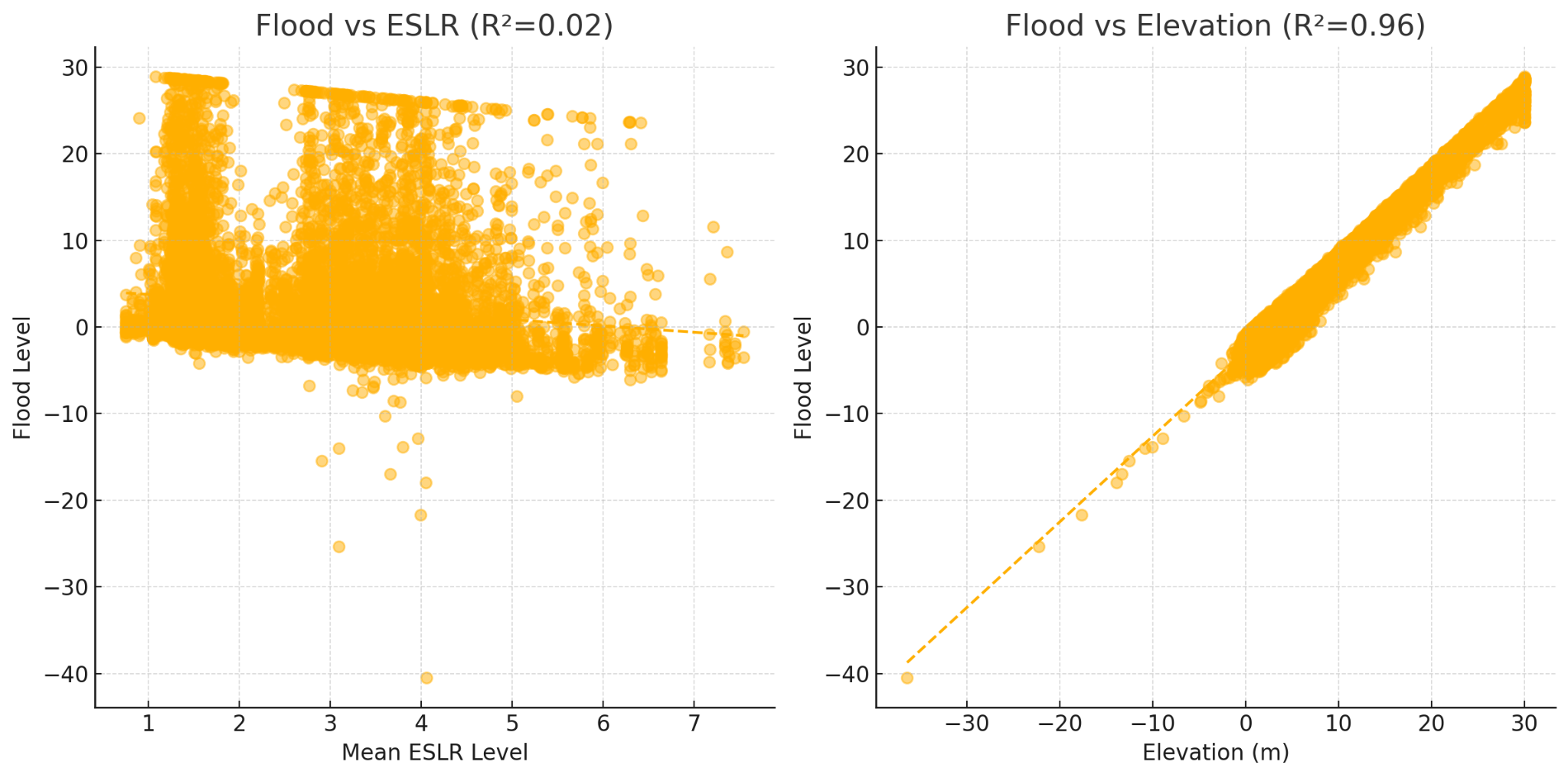
**
